# A Systematical Rheological Study of Maize Kernel

**DOI:** 10.3390/foods12040738

**Published:** 2023-02-08

**Authors:** Shaoyang Sheng, Aimin Shi, Junjie Xing

**Affiliations:** 1 School of Public Health, Anhui Medical University, Hefei 230032, China; 2 Institute of Food Science and Technology, Chinese Academy of Agricultural Sciences, Key Laboratory of Agro-Products Processing, Ministry of Agriculture and Rural Affairs, Beijing 100193, China; 3 State Key Laboratory of Food Science and Technology, School of Food Science and Technology, Jiangnan University, Wuxi 214122, China

**Keywords:** rheological properties, time-temperature superposition principle, maize kernel

## Abstract

In this study, the rheological behavior of maize kernel was systematically investigated using a dynamic mechanical analyzer. The loss in toughness caused by drying resulted in a downward shift in the relaxation curve and an upward shift in the creep curve. The long relaxation behavior became obvious when the temperature was above 45 °C, resulting from the weakening of hydrogen bonds with temperature. The maize kernel relaxed more rapidly at high temperatures, caused by a reduction in the cell wall viscosity and polysaccharide tangles. The Deborah numbers were all much smaller than one, suggesting that the Maxwell elements showed viscous behavior. Maize kernel, as a viscoelastic material, showed a dominant viscous property at high temperatures. The decline in *β* with increasing drying temperature indicated an increase in the width of the relaxation spectrum. A Hookean spring elastic portion made up the majority of the maize kernel creep strain. The order–disorder transformation zone of maize kernel was about 50–60 °C. Due to the complexity of maize kernel, the William–Landel–Ferry constants differed from the universal values; these constants should be ascertained through experiments. Time-temperature superposition was successfully used to describe the rheological behavior. The results show that maize kernel is a thermorheologically simple material. The data acquired in this study can be used for maize processing and storage.

## 1. Introduction

As an important staple crop, maize is widely grown throughout the world [1]. The water content of maize at harvest ranges from 20% to 28% [2]. For safe storage, maize should be dried to a 12–13% water content [3].

Maize can be dried by many methods, such as hot air drying, vacuum drying, solar drying, and microwave drying. Hot air drying is a procedure in which a product is dried with heated air. This drying method is simple and thus commonly used [4]. However, its efficiency is low [5]. In vacuum drying, the product is dried at sub-atmospheric pressure. Products dried using this method are generally of a better quality [6]. The synergistic combination of vacuum and hot air drying results in high-quality products with a short processing time and low power consumption. Maize can be effectively dried using a combination of the vacuum and hot air processes [7]. 

Foods are viscoelastic materials [8]. The data on viscoelastic behavior of foods can be used to describe processing operations [9]. The drying conditions, maize varieties, and ripening stage can affect the viscoelastic properties of maize kernel. Stress relaxation is an essential characteristic of a viscoelastic material [10]. In a stress relaxation experiment, a constant deformation is applied to the product and the decline in the stress over time to keep this deformation is measured [11]. Li et al. [12] found that the five-element Maxwell equation could predict the stress relaxation property of sweet potato better than the three-element Maxwell equation. Wang et al. [11] studied the stress relaxation property of rice. They measured the relaxation modulus. Abedi and Takhar [13] investigated the stress relaxation behavior of banana. The ripening stage could affect the rheological behavior pattern of banana in the drying process.

Creep is another essential characteristic of a viscoelastic material [10]. In a creep experiment, a fixed force is applied to the product and the displacement is measured. Li et al. [12] performed the creep-recovery cycle test on sweet potato. They repeated the cycle three times. After the first cycle, no significant differences in the creep elements were observed. Ditudompo et al. [14] found that the Burgers’ equation could describe the creep behavior of an extruded cornstarch well (*R*^2^ > 0.92). Ozturk and Takhar [15] studied the viscoelastic behavior of carrot. The creep property of carrot was highly correlated with the moisture content. The storage of maize can be simulated by creep experiments [16]. However, it is difficult to conduct stress relaxation and creep experiments over a wide time range.

Prolonging the time and raising the temperature have an equivalent influence on the viscoelastic behavior of materials. This is known as the theory of time–temperature superposition (TTS) [17]. The applicable standards for TTS are as follows [18]: (a) neighboring curves can be joined to form a smooth curve; (b) all the viscoelastic functions must be superimposed with an identical *α_T_*; and (c) the relationship between *α_T_* and temperature must satisfy the empirical equations. Polymers that follow TTS are called thermorheologically simple polymers; those that do not follow TTS are called thermorheologically complex polymers [19]. TTS has previously been tested in various polymers [11,20,21,22,23,24,25]. However, the application of TTS to maize kernel has not been reported.

The purposes of the current study were (i) to describe the viscoelastic behavior of maize kernel dried using a combination of vacuum and hot air processes; (ii) to assess the applicability of TTS to maize kernel; and (iii) to characterize the viscoelastic behavior of maize kernel over a wide timescale using TTS.

## 2. Materials and Methods

### 2.1. Materials

The variety of maize employed in this study was Nongda 86. Its seed kernels were obtained from freshly harvested maize (Beijing Sinong Seed Co., Ltd., Beijing, China) from Zhangye city, Gansu province, China. The kernels’ initial water content was 27%. Prior to further testing, the kernels were kept in self-sealing bags and refrigerated at 4 °C.

### 2.2. Drying of Maize Kernels

The maize kernels were dried using the two methods detailed below. The samples’ water content was determined using a GTR800E single-kernel moisture tester (Shizuoka Seiki Co., Ltd., Shizuoka, Japan).

#### 2.2.1. Natural Drying (ND)

The maize kernels were placed on six glass plates (diameter: 20 cm) (Lanyi reagent company, Beijing, China) in one or two (thin) layers and then dried indoors to a water content of 13%. Finally, the samples were kept in self-sealing bags and refrigerated at 4 °C.

#### 2.2.2. Hot Air/Vacuum Drying (HVD)

In a 50 °C oven (Shanghai Yiheng Scientific Instrument Co., Ltd., Shanghai, China), the maize kernels were firstly dried to a water content of 18%. Then, in a −0.1 MPa DZ-3 vacuum drier (Taisite Instrument Co. Ltd., Tianjin, China), the samples were separately dried at 75 °C, 65 °C, 55 °C, 45 °C, and 35 °C to a water content of 13%. Finally, the samples were kept in self-sealing bags and refrigerated at 4 °C.

### 2.3. Sample Preparation

To ensure the maize kernels had a flat and smooth surface, every sample was carefully polished by hand to prevent any physical failure caused by using abrasive papers. A Fowler PRO-MAX digital vernier caliper (Newton, MA, USA) with a precision of 0.01 mm was employed to determine the width and length of every sample. The dimensions of the samples were about 9 mm × 4 mm × 4 mm (length × width × height). The sample preparation process was based on our previous research [26].

### 2.4. Stress Relaxation Tests

A TA Instruments Q800 dynamic mechanical analyzer (DMA, New Castle, DE, USA) was employed to measure the maize kernels’ relaxation moduli. The compression mode was employed to carry out the tests. A 0.05 N preload was applied to ensure complete contact between the surface of the sample and the compression plate. The samples were equilibrated at four test temperatures (85 °C, 65 °C, 45 °C, and 25 °C) for 1 min. Then, the tests were conducted at the 0.8% strain for 10 min.

The generalized Maxwell equation (Equation (1)) was employed to describe the maize kernel relaxation modulus:

(1) $$E(t)=\sum_{m=1}^{n}E_{m}\exp(-t/\tau_{m})+E_{0}$$

where *E*(*t*) (MPa) and *E*_0_ (MPa) denote the stress relaxation modulus and equilibrium modulus, respectively; *τ_m_* (s) is the relaxation time; *E_m_* (MPa) is the corresponding relaxation modulus; and *t* (s) is the time.

The Kohlrausch–Williams–Watts (KWW) equation (Equation (2)) was also employed to predict the modulus:

(2) $$E(t)=E_{0}\exp[-{\left(t/\tau \right)}^{\beta }]$$

where *E*_0_ (MPa) denotes the initial modulus, *τ* (s) denotes the relaxation time, and *β* denotes the exponent.

### 2.5. Creep Tests

The DMA was also employed to determine the kernels’ strains. The compression mode was employed to carry out the tests. A 0.05 N preload was supplied to each kernel. The samples were equilibrated at four test temperatures (60 °C, 50 °C, 40 °C, and 30 °C) for 1 min. Then, the tests were performed at 0.08 MPa stress for 10 min.

Burgers’ equation (Equation (3)) was employed to predict the creep strain.

(3) $$\varepsilon =\frac{\sigma }{E_{1}}+\frac{\sigma }{E_{2}}\left[1-\exp\left(-t\frac{E_{2}}{\eta_{2}}\right)\right]+\frac{\sigma }{\eta_{1}}t$$

In Equation (3), *t* (s) represents time; *ε* (dimensionless) denotes the kernel’s strain; *σ* (MPa) denotes the applied stress; *η*_1_ (MPa s) and *η*_2_ (MPa s) denote the Maxwell dashpot viscosity and Kelvin dashpot viscosity, respectively; and *E*_1_ (MPa) and *E*_2_ (MPa) denote the Maxwell spring modulus and Kelvin spring modulus, respectively. Generally, *η*_2_/*E*_2_ = *τ*, where *τ* denotes the Kelvin element retardation time.

From the differentiation of Equation (3), the creep rate *ε*’ can be acquired:

(4) $$\varepsilon ’=\frac{d\varepsilon }{dt}=\frac{\sigma }{\eta_{1}}+\frac{\sigma }{\eta_{2}}\exp\left(-t\frac{E_{2}}{\eta_{2}}\right)$$

When the time tends to infinity, *ε*’ will gradually reach a certain value:

(5) $$\varepsilon ’(\infty )=\frac{d\varepsilon }{dt}{|}_{t=\infty }=\frac{\sigma }{\eta_{1}}$$

Another equation, called the Findley power law, can also describe the strain of polymers [27]. It has the following form:

(6) $$\varepsilon =at^{b}+\varepsilon_{0}$$

Here, *ε*_0_ denotes the initial deformation, while *a* and *b* denote the material parameters.

A simpler two-parameter power law equation has also been employed to describe the strain of the polymers [28,29,30]:

(7) $$\varepsilon =at^{b}$$

### 2.6. Time–Temperature Superposition

The strain and relaxation modulus of the maize kernels over a wide time range were described using TTS. The master curves were produced by transferring the short-period curves horizontally [31]. The William–Landel–Ferry (WLF) [19] (Equation (8)) or Arrhenius equation [32] (Equation (9)) can be employed to describe the shift factor.

(8) $$\log\alpha_{T}=\frac{C_{1}T_{0}-C_{1}T}{T-T_{0}+C_{2}}$$

In Equation (8), *α_T_*, *T*_0_ (K), and *T* (K) represent the shift factor, reference temperature, and test temperature, respectively, while *C*_1_ and *C*_2_ (K) are the WLF parameters.

(9) $$\log\alpha_{T}=\frac{E_{a}}{R}(\frac{1}{T_{}}-\frac{1}{T_{0}})$$

In Equation (9), *R* (J/K·mol) and *E_a_* (kJ/mol) denote the gas law constant and activation energy, respectively, while *T*_0_ (K) and *T* (K) denote the reference and experimental temperatures, respectively.

As suggested by Ferry [18], the Arrhenius equation and WLF equation were both employed to describe *α_T_*. *C*_1_, *C*_2_, *E_a_*, and the coefficient of determination (*R*^2^) were determined from the experimental data using regression analysis. Temperatures of 25 °C and 30 °C were chosen as the reference temperatures for stress relaxation and creep TTS, respectively.

### 2.7. Statistical Analysis

Three replicates were conducted for every sample. Universal Analysis 2000 software (version 4.3A, TA Instruments, New Castle, DE, USA) was employed to acquire the DMA data. SPSS 17.0 (SPSS Inc., Chicago, IL, USA) was employed to conduct the statistical analysis.

## 3. Results and Discussion

### 3.1. Analysis of Stress Relaxation Behavior

**Figure 1:**
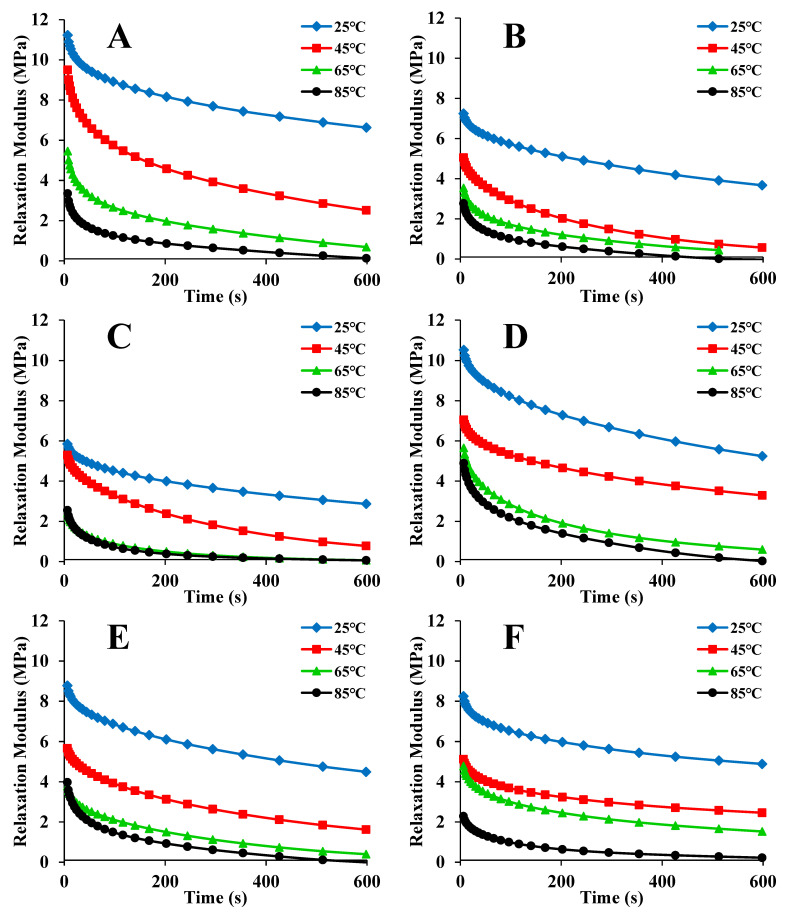
Relaxation modulus data for maize kernels at different temperatures: (**A**) natural drying (ND); (**B**) hot air/vacuum drying (HVD) 35 °C; (**C**) HVD 45 °C; (**D**) HVD 55 °C; (**E**) HVD 65 °C; (**F**) HVD 75 °C.

The stress relaxation data for maize kernels at different temperatures are plotted in Figure 1. Three stages make up the curve. The first stage shows an immediate elastic response. The timescale of this region is nearly zero. The second stage demonstrates a marked stress relaxation process. The timescale is from about 0 to 20 s. In the third stage, the modulus approaches a constant. The maize kernel is viscoelastic [8]. When strain is applied to the kernel, the stress increases immediately due to the elastic portion of the kernel and then decreases gradually due to the viscous portion of the kernel. This trend is identical to the findings reported in a previous study [33]. It can be seen from Figure 1 that the kernels dried by ND had the highest initial relaxation modulus. This is because the kernels dried by ND had the highest toughness. For the same sample, when the temperature was increased, the initial modulus declined markedly. This was caused by an increase in the motion of the polymer chains, resulting in a lowering of the threshold for specific strain conditions [34].

Table 1 presents the elements of the generalized Maxwell equation. As listed in Table 1, the *R*^2^ was around 0.94–0.98, indicating that the stress relaxation modulus was described well by the generalized Maxwell equation. A similar finding was reported in a study on oat grains [35]. Table 1 also shows that *E*_1_ decreased as the temperature increased, suggesting that higher temperatures reduced the maize kernel’s deformation resistance [36]. Hence, the stress required to achieve the given strain decreased. At the same temperature, *τ* fluctuated with drying conditions and did not display a decreasing or increasing tendency, suggesting that the relaxation time was not related to the drying method. In general, *τ* decreased as the temperature increased. That is, kernels relaxed more rapidly at higher temperatures. The reason for this is as follows: when a maize kernel becomes soft due to high temperatures, the cell wall viscosity and polysaccharide tangles will be reduced, leading to faster stress dissipation [36]. By using the expression below, the Deborah number (*D_e_*) can be obtained:

(10) $$D_{e}=\tau /t$$

Here, *t* denotes the observation time. *D_e_* can provide useful information on materials’ viscoelastic properties: *D_e_* ≈ 1 denotes a viscoelastic behavior; *D_e_* << 1 denotes a viscous fluid; and *D_e_* >> 1 denotes an elastic solid [37]. The *D_e_* values (0.094–0.385) calculated in this study were much smaller than one; this suggests that the Maxwell element showed viscous behavior.

Table 1 also shows that when the temperature increased, *E*_0_ decreased. This suggests that maize kernel is mainly a viscous material at high temperatures. The maize kernel’s viscous portion begins to decrease as the temperature decreases. According to the theory of free volume, molecular dynamics can account for the influence of the viscous portion. When the temperature is low, there is not enough free volume to allow for the motion of molecular chains; thus, the molecules’ movement is fixed. This makes the kernel glassy. When the temperature is high, the free volume becomes larger and the molecular chains can move. This makes the kernel rubbery [11]. In addition, the *E*_0_ for ND was higher than the *E*_0_ for HVD (at the same temperature), except in the case of HVD 55 °C at 45 °C and HVD 75 °C at 65 °C. This suggests that the kernel’s stiffness changed. This is owing to the changes in the kernel’s cellular structure caused by hot air/vacuum drying, giving rise to a decline in the stiffness. The change in *E*_0_ may have resulted from the breakdown of cell membranes caused by HVD [38], leading to the collapse of the cells [33,39]. 

### 3.2. Analysis of Creep Behavior

**Figure 2:**
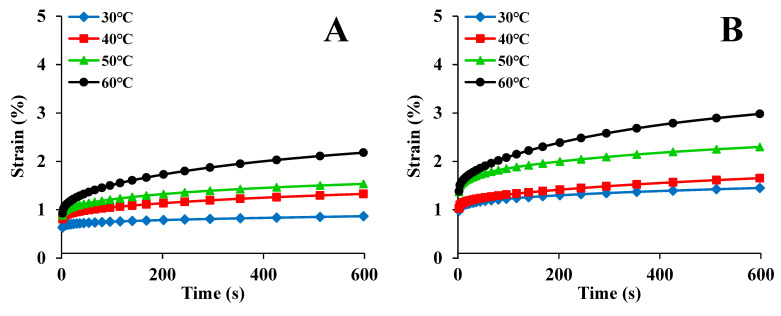

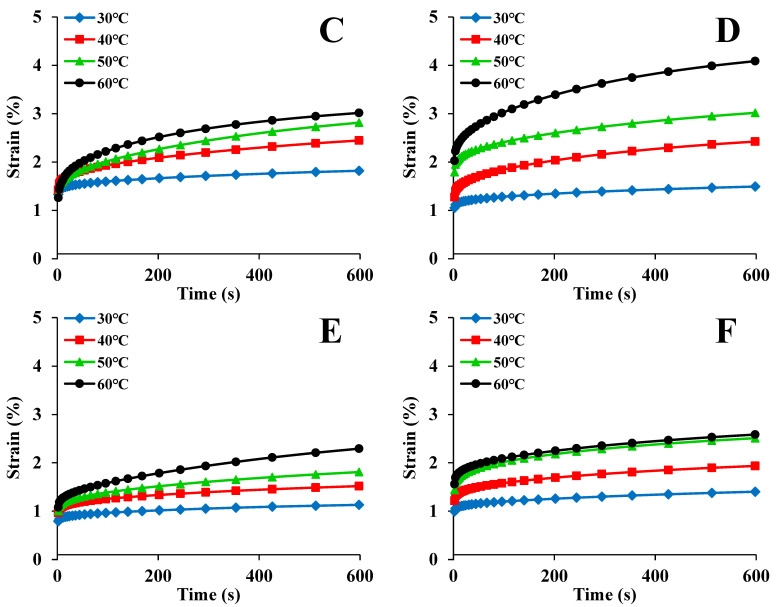
Creep data for maize kernels at different temperatures: (**A**) ND; (**B**) HVD 35 °C; (**C**) HVD 45 °C; (**D**) HVD 55 °C; (**E**) HVD 65 °C; (**F**) HVD 75 °C.

Figure 2 displays the creep curves for maize kernels at various temperatures. The curves demonstrate classic creep features and exhibit a similar tendency over time. When stress is applied to the maize kernels, the strain increases instantly because of elasticity. In this portion, the changes in the kernel’s structure are reversible [40]. Then, the strain continues to increase at a decreasing rate due to viscosity. This portion of the curve contributes to the maize kernel’s viscoelastic property [15]. The last portion is a straight line, representing the kernel’s viscous flow deformation. In this compression stage, the kernel’s tissues are permanently destroyed, and the changes can only be partially reversed [41]. This tendency is consistent with the creep strain of barley kernels reported in a previous study [42]. In this study, the creep strain exhibited a strong temperature dependence. When the temperature increased, the strain also increased. A similar finding was reported in a previous study [43]. Therefore, the solid-like characteristics of maize kernel increase as the temperature decreases [14]. It can also be seen from Figure 2 that the ND kernels had the lowest creep strain. This is because the kernels dried using the ND method were more compact and robust.

Table 2 displays the values of the four elements (*E*_1_, *E*_2_, *η*_1_, and *η*_2_) mentioned in Section 2.5. The data in this table suggest that Burgers’ equation can predict the creep property well (*R*^2^ > 0.98). *E*_1_, *E*_2_, *η*_1_, and *η*_2_ all decreased as the temperature increased, suggesting that increasing temperature increases the deformation of maize kernel. *E*_1_ is correlated with the elasticity of a semi-crystalline polymer’s crystalline regions. In contrast to the amorphous zones, the crystalline regions are exposed to instant stress because of their relatively high hardness. When the stress is eliminated, *E*_1_ can be resumed instantly [44]. *E*_2_ is correlated with the rigidity of a material [30]. When the temperature increases, the instant and viscoelastic strain increase. As a result, *E*_1_ and *E*_2_ decrease. *η_1_* is correlated with the damage from the crystalline regions and the irrevocable strain from the amorphous zones. *η_2_* corresponds to the viscosity of the semi-crystalline polymer’s amorphous zones [45]. When the temperature increases, the values of *η*_1_ and *η*_2_ decline. This indicates that the molecular chains become more active [29]. The ND elements exhibited the greatest declines (particularly in *η*_1_, which depends heavily on temperature and is correlated with the rate of long-period deformation), suggesting that the viscoelastic property of kernel dried by ND was more sensitive to temperature. 

It can also be seen from Table 2 that *E*_2_ was larger than *E*_1_. In comparison to the viscous and retarded elastic strain, the instant strain was larger. Thus, the Hookean spring elastic portion mainly makes up maize kernel’s creep strain [14]. Table 2 also presents *ε*’ (∞) and *τ* for the maize kernels. When the temperature was increased, *ε*’ (∞) increased monotonically. At 60 °C, the kernels generally showed a longer retardation time (*τ*), indicating that the samples’ viscoelastic property remained for longer [41].

Overall, the kernel dried by ND had a higher stiffness. The viscoelastic property of the kernel dried by ND was more sensitive to temperature. The relaxation time of the kernel was not related to the drying method.

### 3.3. Applicability of Time-Temperature Superposition

**Figure 3:**
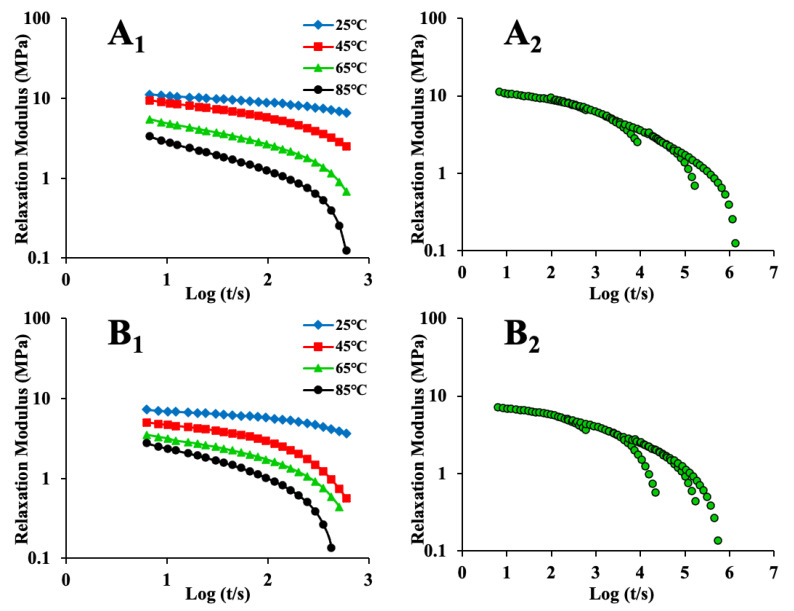

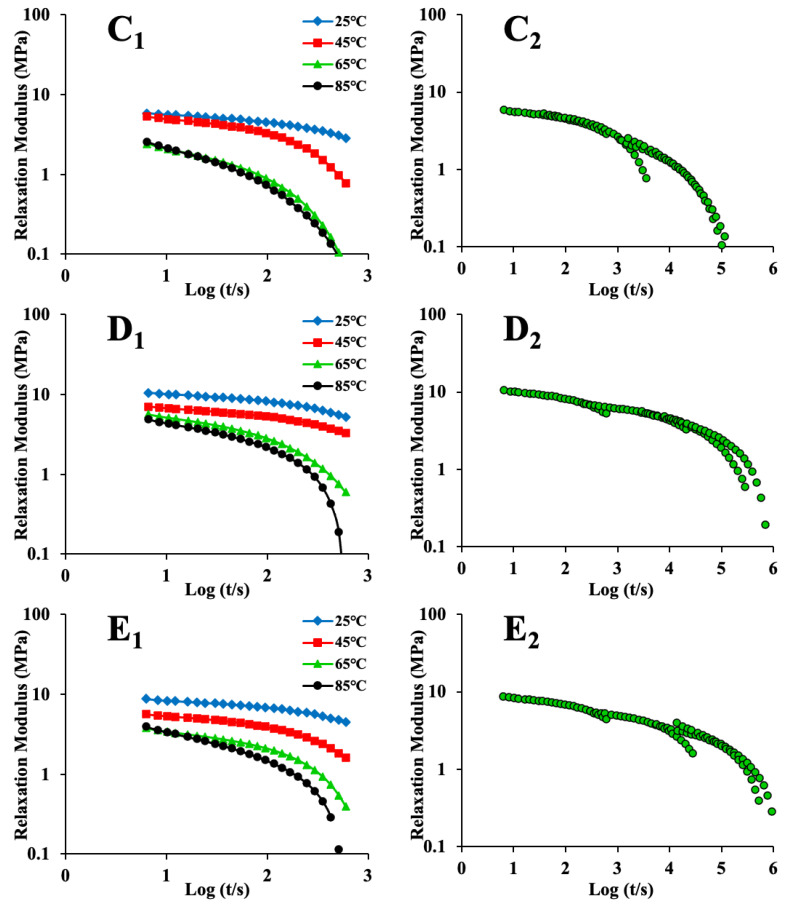

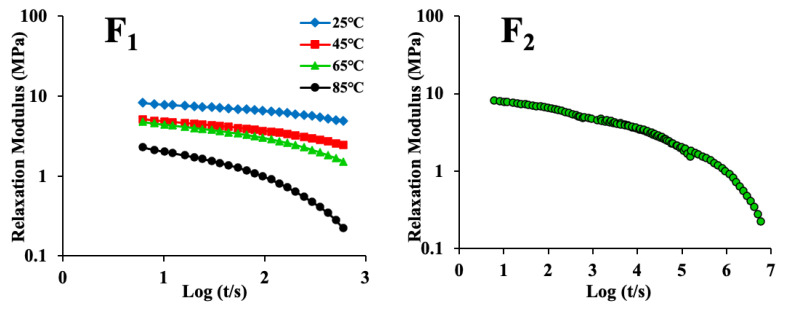
The left-hand graphs show logarithmic plots of stress relaxation data for maize kernels, while the right-hand graphs are the corresponding master curves for maize kernels (**A_1_**,**A_2_**)—ND; (**B_1_**,**B_2_**)—HVD 35 °C; (**C_1_**,**C_2_**)—HVD 45 °C; (**D_1_**,**D_2_**)—HVD 55 °C; (**E_1_**,**E_2_**)—HVD 65 °C; (**F_1_**,**F_2_**)—HVD 75 °C).

**Figure 4:**
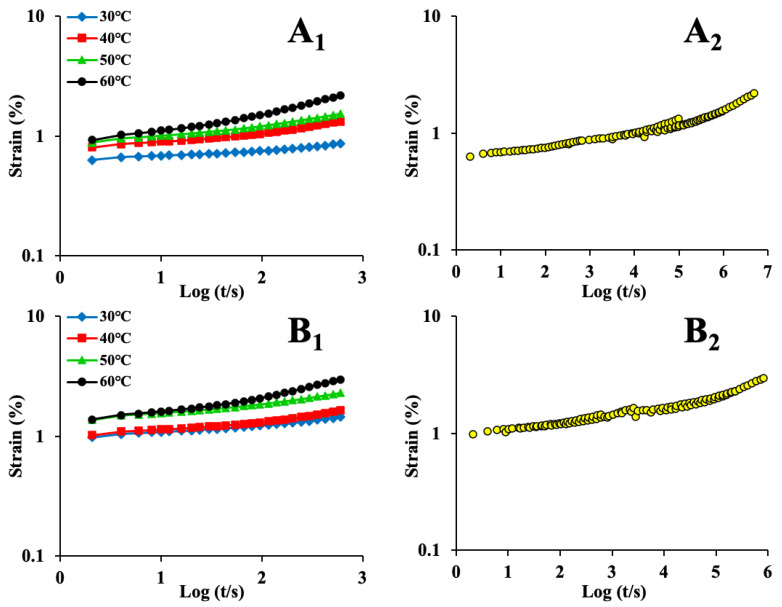

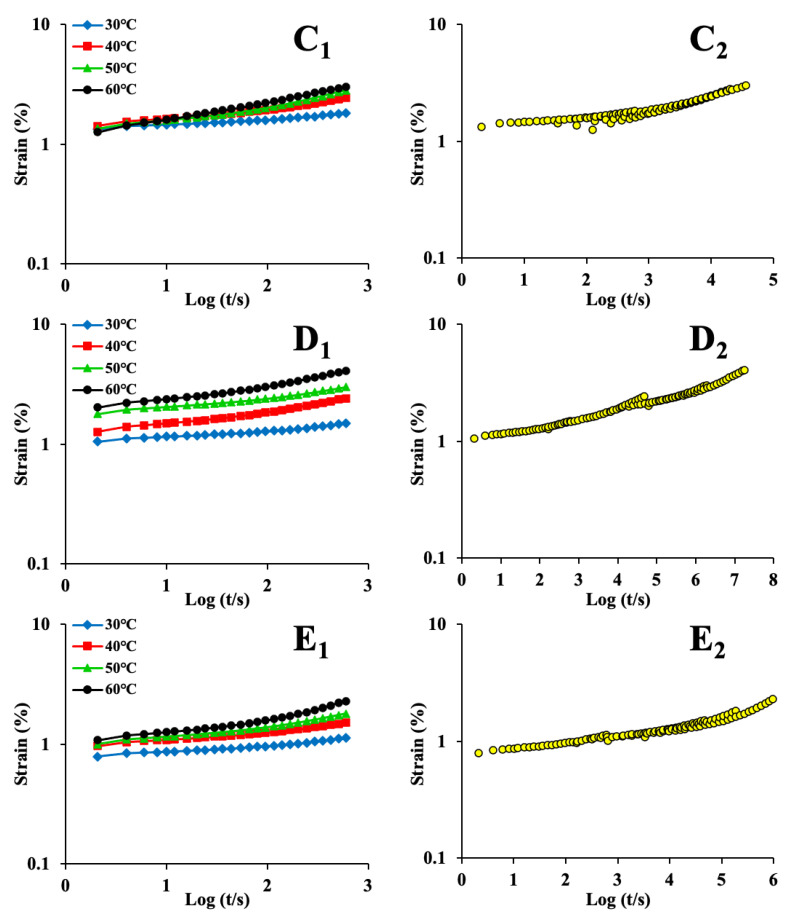

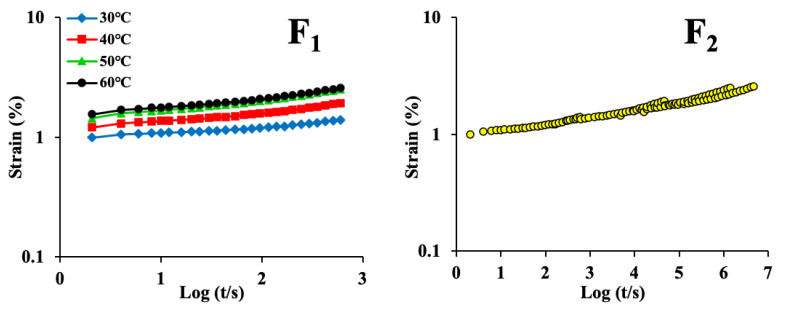
The left-hand graphs show logarithmic plots of creep data for maize kernels, while the right-hand graphs are the corresponding master curves for maize kernels (**A_1_**,**A_2_**)—ND; (**B_1_**,**B_2_**)—HVD 35 °C; (**C_1_**,**C_2_**)—HVD 45 °C; (**D_1_**,**D_2_**)—HVD 55 °C; (**E_1_**,**E_2_**)—HVD 65 °C; (**F_1_**,**F_2_**)—HVD 75 °C.

Figure 1 and Figure 2 were replotted in logarithmic form (left-hand graphs in Figure 3 and Figure 4) to employ TTS. It can be seen that for stress relaxation, the impact of temperature was obvious at 45 °C and became more significant at 65 °C; for creep, the impact was obvious at 50 °C and became significant at 60 °C. The temperature range of 45–65 °C and 50–60 °C denoted the order–disorder transformation zones of maize kernel [46]. These are close to the gelatinization temperature of maize starch (64–72 °C) [47]. Furthermore, from the graphs on the left in Figure 3, it can be seen that the long relaxation behavior became obvious when the temperature was above 45 °C. Specific molecular interactions in maize kernel and temperature have an impact on when the long relaxation process occurs [21]. In general, in this study, long relaxation occurred when the time was approximately 200 s at 65 °C, and when it was 160 s at 85 °C. This is because the hydrogen bonds were weakened when the temperature was raised [48].

By shifting the stress relaxation (left-hand graphs in Figure 3) and creep (left-hand graphs in Figure 4) curves horizontally, the master curves were acquired (right-hand graphs in Figure 3 and Figure 4). As noted earlier, the applicable standards for TTS are as follows: (a) neighboring curves can be joined to form a smooth curve; (b) all the viscoelastic functions must be superimposed with an identical *α_T_*; and (c) the relationship between *α_T_* and the temperature must satisfy the empirical equations. It can be seen from the graphs in Figure 3 and Figure 4 (right) that the relaxation and creep curves exhibited superposition for all the samples, although the master curves showed some tails and irregularities (Figure 3(A_2_–E_2_) and Figure 4(B_2_,C_2_)). The absence of superposition is correlated with the relaxation mechanism, which depends on temperature and takes place inside the maize kernel [49]. This is parallel to the findings reported in a previous study [50]. In that study, the curves obtained at higher temperatures could not be overlapped smoothly with only horizontal shifting. Nevertheless, by shifting the obtained curves both vertically and horizontally, smooth master curves were acquired. In general, the relaxation modulus and creep strain master curves were considered to be continuous and smooth in this study. Some curves (Figure 3(F_2_) and Figure 4(A_2_,D_2_–F_2_)) demonstrated an excellent fit for the TTS. These findings suggest that the maize kernel is a thermorheologically simple material [51]. The applicability of TTS has also been evaluated for other food materials [46,49,52,53,54], and soybean [55] was reported as a thermorheologically simple material.

**Figure 5:**
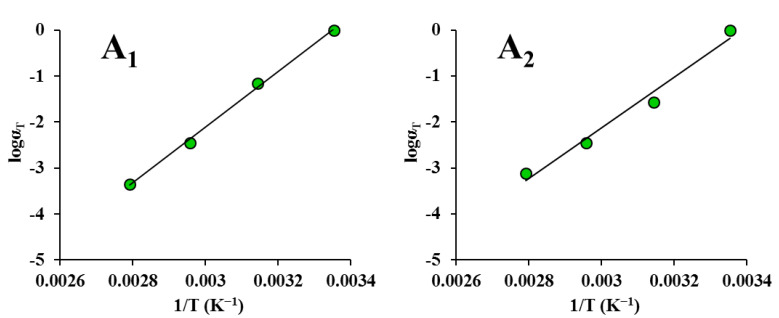

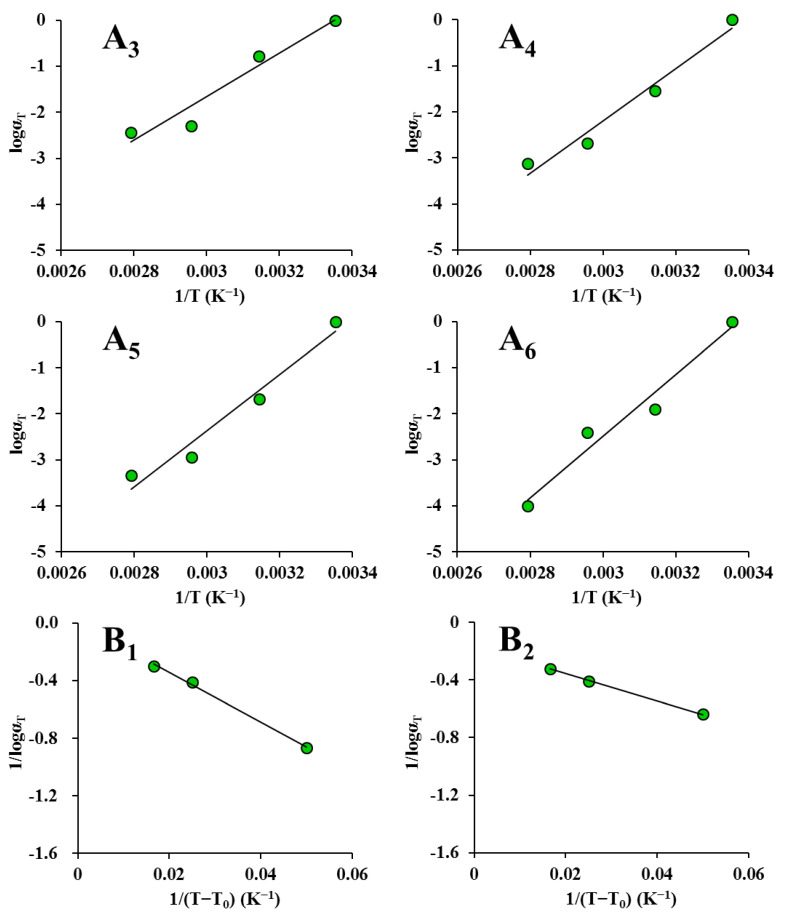

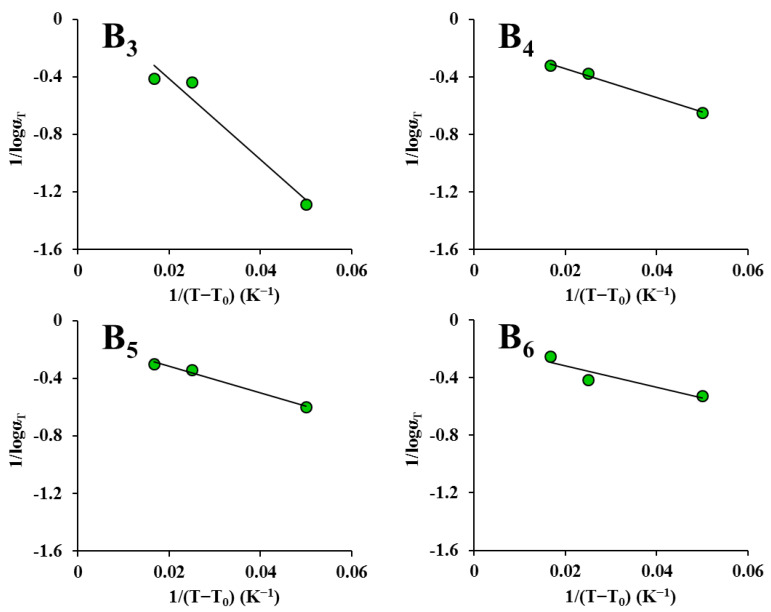
Shift factors fitted to the Arrhenius equation for maize kernels (stress relaxation): (**A_1_**) ND; (**A_2_**) HVD 35 °C; (**A_3_**) HVD 45 °C; (**A_4_**) HVD 55 °C; (**A_5_**) HVD 65 °C; (**A_6_**) HVD 75 °C. Shift factors fitted to the William–Landel–Ferry (WLF) equation for maize kernels (stress relaxation): (**B_1_**) ND; (**B_2_**) HVD 35 °C; (**B_3_**) HVD 45 °C; (**B_4_**) HVD 55 °C; (**B_5_**) HVD 65 °C; (**B_6_**) HVD 75 °C.

**Figure 6:**
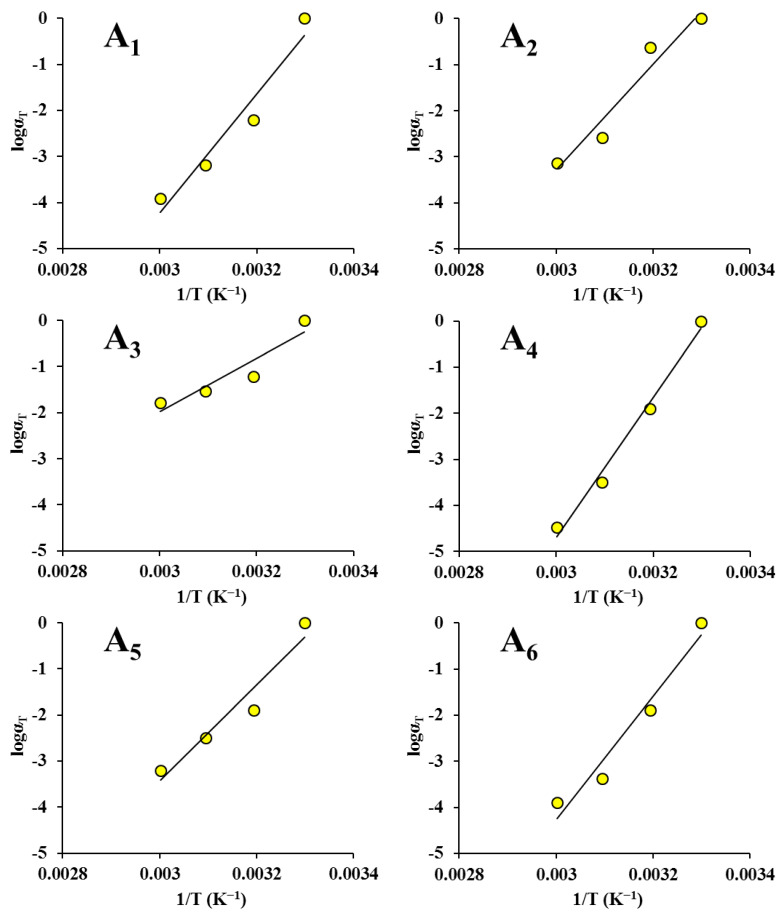

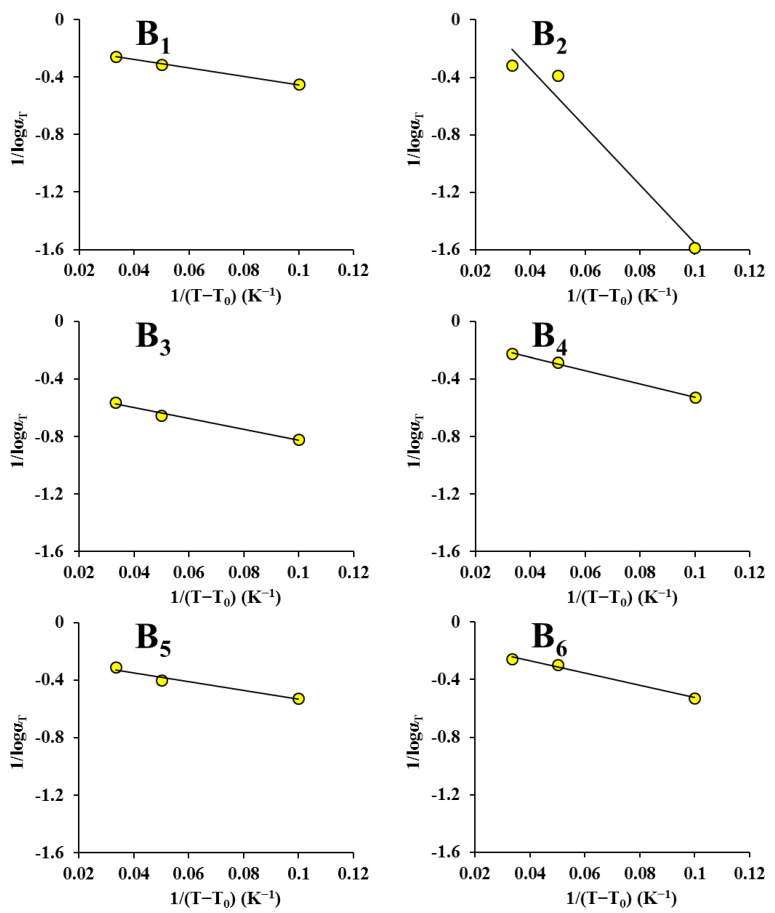
Shift factors fitted to the Arrhenius equation for maize kernels (creep): (**A_1_**) ND; (**A_2_**) HVD 35 °C; (**A_3_**) HVD 45 °C; (**A_4_**) HVD 55 °C; (**A_5_**) HVD 65 °C; (**A_6_**) HVD 75 °C. Shift factors fitted to the WLF equation for maize kernels (creep): (**B_1_**) ND; (**B_2_**) HVD 35 °C; (**B_3_**) HVD 45 °C; (**B_4_**) HVD 55 °C; (**B_5_**) HVD 65 °C; (**B_6_**) HVD 75 °C.

It can be seen from Figure 5 and Figure 6 that *α_T_* reduced monotonically with the temperature. This is similar to the finding of Meza et al. [56]. Furthermore, *α_T_* versus the temperature data could be fitted well by both the Arrhenius equation and the WLF equation (*R*^2^ > 0.857). However, the correlation between *α_T_* and the temperature was not very high for some samples. This is because the temperatures were too close, and no remarkable viscoelastic variations were detected, similar to the finding reported by Ahmed [49]. In his study, no noticeable rheological variations were found in guar gum dispersions due to the closeness of the temperatures.

Table 3 and Table 4 display the Arrhenius activation energy (*E_a_*) and WLF constants. The WLF constants, *C*_1_ and *C*_2_, are given by [57]:

(11) $$C_{1}=\frac{B}{2.303f}$$

(12) $$C_{2}=\frac{f}{\alpha }$$

Here, *B* can be taken equal to unity, *f* denotes the fractional free volume at the reference temperature, and *α* denotes the thermal expansion coefficient. Although the WLF constants fluctuated with the drying conditions, the WLF constants for ND and HVD 35 °C were the highest, suggesting that the fractional free volume and thermal expansion coefficient were the smallest.

It can be seen that the *R*^2^ of the WLF equation was higher than that of the Arrhenius equation. A similar finding was reported in a previous study [58]. Despite this, it should be noted that there are two coefficients in the WLF equation, while there is only one coefficient in the Arrhenius equation. Hence, the WLF equation is more adaptable. We can also observe from Table 3 and Table 4 that the *C*_1_ and *C*_2_ values ranged from 2.226 to 32.020 and 8.346 to 257.500 K, respectively. The values of *C*_1_ were slightly different from the universal value (*C*_1_ = 17.44), while the values of *C*_2_ were considerably different (*C*_2_ = 51.6 K) [57]. This is due to the complexity of food polymers (including different charges on the surfaces of polymers, various polysaccharides, 20 amino acids of protein, large polydispersities) [59] and is similar to the finding of Altay and Gunasekaran [60]. In their research, the values of *C*_1_ and *C*_2_ for a gelatin–xanthan gum system ranged from 4.52 to 22.68 and 58.97 to 204.97 K, respectively. Peleg [61] stated that employing universal values in food polymers ought to be approached cautiously. The values of *C*_1_ and *C*_2_ for food materials should be ascertained through experiments due to their substance-specific characteristics [59]. 

In this study, the *E_a_* values for stress relaxation ranged from 90.86 to 134.8 kJ/mol. These values are very close to the *E_a_* value (94.3 kJ/mol) of rice kernels (13.8% moisture content) for stress relaxation [11]. This is likely because maize kernels and rice kernels are both mainly composed of starch. The values for creep ranged from 111.035 to 292.645 kJ/mol, which are higher than the values (62.960–166.539 kJ/mol) for starch films [62,63]. Furthermore, the values for creep were significantly higher than those for stress relaxation. The *E_a_* for relaxation and creep represents the energy needed for the initiation of the molecular movements, leading to relaxation and creep [64]. From Table 3 and Table 4, the values for the kernels dried by HVD at 75 °C and HVD at 55 °C were the highest, suggesting that more energy was required, while the values for the kernels dried by HVD at 45 °C were the lowest.

**Figure 7:**
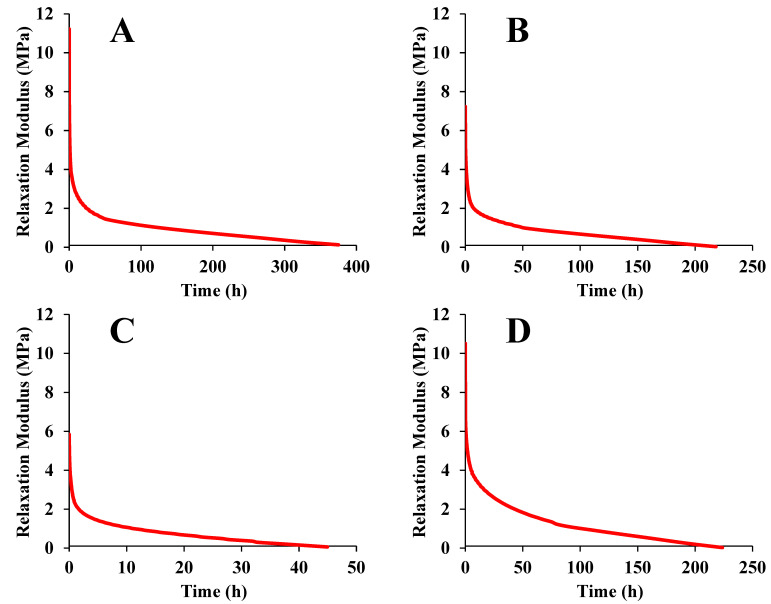

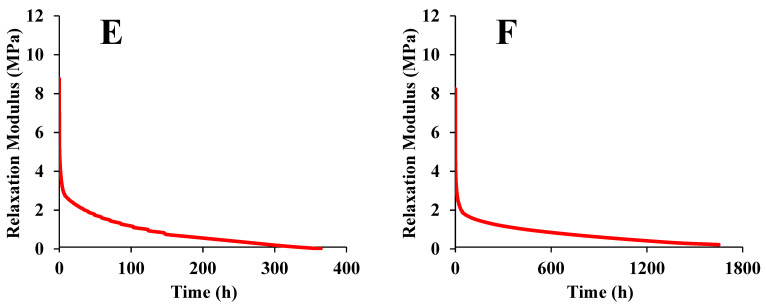
Time-temperature superposition (TTS) predicted stress relaxation for maize kernels dried under different conditions: (**A**) ND; (**B**) HVD 35 °C; (**C**) HVD 45 °C; (**D**) HVD 55 °C; (**E**) HVD 65 °C; (**F**) HVD 75 °C.

Overall, TTS was successfully applied to the maize kernel. TTS extended the timescale of the relaxation modulus and creep strain from 2 to 5–6 log periods (Figure 3) and 2 to 4–7 log periods (Figure 4), respectively. Performing a DMA experiment for such a long time is extremely challenging. The application of TTS to the rice kernel was reported in a previous study [11]. In that study, the timescale of the relaxation modulus for the rice kernel was extended from 2 to 6 log periods.

### 3.4. Long-Period Stress Relaxation and Creep Response

Figure 7 shows the TTS predicted relaxation moduli for maize kernels dried under different conditions at 25 °C. As shown in this figure, the resulting predicted curves provide an accelerated stress relaxation characterization up to 1600 h. Constant strain-rate tests performed for yellow-dent maize kernels were reported in a previous study [9]. In that study, the timescale of the relaxation modulus was extended to 10^6^ s; however, the modulus was higher than that in the current study. This may have resulted from differences in the maize varieties and experimental conditions.

**Figure 8:**
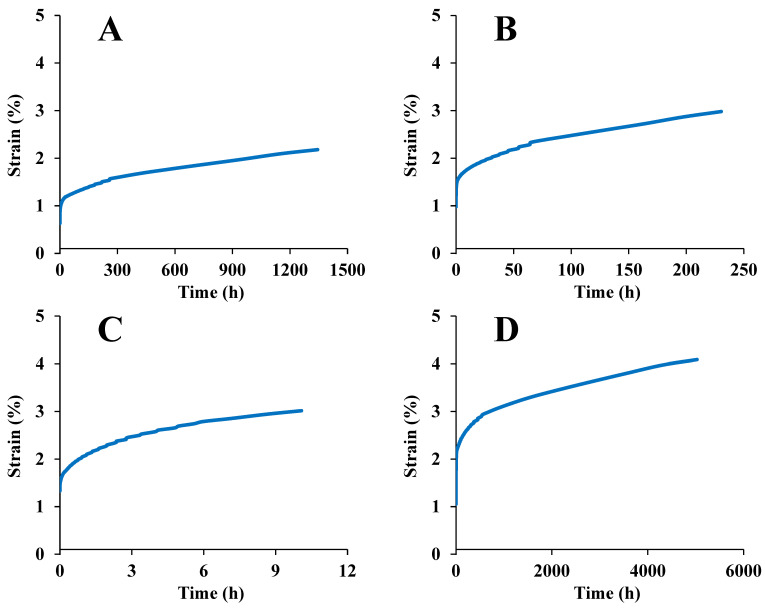

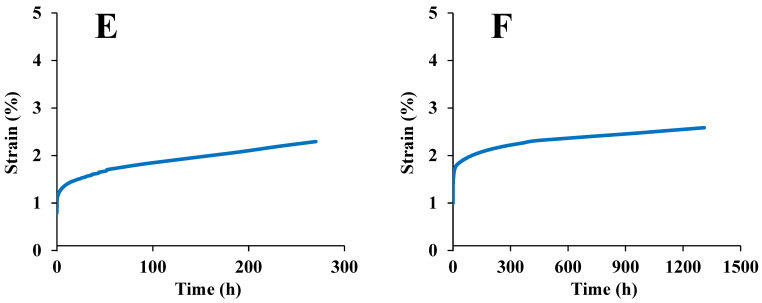
TTS predicted creep for maize kernels dried under different conditions: (**A**) ND; (**B**) HVD 35 °C; (**C**) HVD 45 °C; (**D**) HVD 55 °C; (**E**) HVD 65 °C; (**F**) HVD 75 °C.

Figure 8 shows the TTS predicted creep strains for maize kernels dried under different conditions at 30 °C. The timescale of the creep strain was extended to 5000 h, and the trends of the curves were similar in the longer time frame. The timescale of the creep strain for the starch film was extended to 10^7^ s in another study [63].

Table 5 lists the equation parameters for long-period stress relaxation. We can observe from this table that both the Maxwell equation and KWW equation fitted the data well. The *R*^2^ of the Maxwell equation was slightly higher than that of the KWW equation. Similar studies have reported fitting the generalized Maxwell equation to the relaxation modulus data of corn kernels [9] and rice kernels [11]. In this study, the values of *E*_1_, *E*_2_, and *E*_3_ were close to each other. This suggests that the maize kernel’s inner structure changed slightly during the long-period relaxation [36]. Furthermore, for the entire relaxation time, the Deborah numbers (*D_e_*_1_ = 0.00034–0.14918; *D_e_*_2_ = 0.00004–0.21343; *D_e_*_3_ = 0.00011–0.06139) were much smaller than one. This suggests that the first, second, and third Maxwell elements all show a viscous response.

The value of the exponent *β* declined from 0.194 to 0.145 when the drying temperature was increased to 75 °C, except in the case of HVD at 45 °C. In accordance with Ngai et al.’s coupling theory [65], the medium and the relaxing species become more tightly coupled when *β* declines. This is correlated with overall declines in the molecular motion and increases in the width of the relaxation spectrum. The decline in *β* results from the increase in the density of the polymer with increasing drying temperature. When the space among the molecules is reduced, the motion of molecular chains is more limited, increasing the width of the relaxation spectrum [66].

Table 6 lists the equation parameters for long-period creep. We can observe from this table that the creep data were described well by Burgers’ equation for all the maize kernels. Nonetheless, long-term experiments have considerably different elements than short-term experiments, caused by fitting a higher rate of creep to the strain in the short-term experiments [29].

The Findley power law equation can better represent the TTS predicted strain than Burgers’ equation. The two-parameter power law equation also describes the creep data well. When the parameter *ε*_0_ was removed, *a* and *b* became more consistent. This is similar to the finding from a previous study [19].

## 4. Conclusions

With regard to stress relaxation, creep, and TTS, the rheological properties of maize kernels dried using a combination of vacuum and hot air processes were investigated. The kernel dried by ND had a higher stiffness. The viscoelastic property of kernel dried by ND was more sensitive to temperature. The relaxation time of the kernel was not related to the drying method. The long relaxation behavior became obvious when the temperature was above 45 °C, as a result of the weakening of hydrogen bonds with temperature. The *D_e_*s were all much smaller than one, indicating that the Maxwell elements showed viscous behavior. The maize kernels exhibited dominant viscous behavior at high temperatures. The decline in *β* with increasing drying temperature indicates an increase in the width of the relaxation spectrum. The order–disorder transformation zone of maize kernel was about 50–60 °C. Finally, TTS was successfully applied to predict the long-period relaxation modulus (up to 1600 h) and creep strain (up to 5000 h). These results show that maize kernel is a thermorheologically simple material. Further structural analyses (such as atomic force microscope and nuclear magnetic resonance analyses) are needed to clarify the molecular mechanism of the HVD kernels. The data acquired in this study can be used for maize processing and storage.

## Figures and Tables

**Table 1:** Parameters of the generalized Maxwell equation for maize kernels.

Drying Condition	*T* (°C)	*E*_1_ (MPa)	*τ* (s)	*E*_0_ (Mpa)	*R* ^2^
ND	25	3.893	155.324	6.894	0.970
	45	5.934	127.648	3.024	0.973
	65	3.855	101.195	1.150	0.960
	85	2.580	70.449	0.502	0.949
HVD 35 °C	25	3.349	231.258	3.609	0.982
	45	4.180	168.697	0.631	0.989
	65	2.265	122.905	0.674	0.971
	85	2.337	87.183	0.203	0.964
HVD 45 °C	25	2.715	198.521	2.915	0.982
	45	4.308	194.858	0.738	0.990
	65	2.045	82.344	0.208	0.977
	85	2.237	56.494	0.225	0.976
HVD 55 °C	25	4.893	211.059	5.252	0.983
	45	3.368	187.663	3.374	0.980
	65	4.495	118.842	0.866	0.984
	85	4.144	119.810	0.359	0.973
HVD 65 °C	25	3.896	206.480	4.516	0.981
	45	3.848	204.399	1.597	0.989
	65	2.994	163.361	0.506	0.980
	85	3.269	86.022	0.364	0.962
HVD 75 °C	25	2.867	154.009	5.058	0.974
	45	2.260	129.410	2.635	0.974
	65	2.791	124.449	1.743	0.976
	85	1.803	87.104	0.367	0.980

**Table 2:** Parameters of Burgers’ equation for maize kernels.

Drying Condition	*T* (°C)	*E*_1_ (MPa)	*E*_2_ (MPa)	*η*_2_ (MPa s)	*η*_1_ (MPa s)	*τ* (s)	*ε*’ (∞) (×10^−6^ s^−1^)	*R* ^2^
ND	30	12.366	93.513	2123.752	3.343 × 10^4^	22.711	2.393	0.986
	40	9.459	44.621	1960.307	1.500 × 10^4^	43.932	5.335	0.991
	50	8.550	29.893	1429.997	1.362 × 10^4^	47.837	5.874	0.991
	60	8.037	15.943	884.383	6667.172	55.472	11.999	0.995
HVD 35 °C	30	7.874	44.399	1249.639	1.758 × 10^4^	28.146	4.551	0.986
	40	7.510	39.770	1249.193	1.169 × 10^4^	31.410	6.846	0.991
	50	5.546	21.809	782.902	9165.199	35.898	8.729	0.989
	60	5.392	12.599	869.341	5301.574	69.001	15.090	0.995
HVD 45 °C	30	5.860	42.303	822.937	1.662 × 10^4^	19.453	4.813	0.982
	40	5.346	21.779	685.719	7719.949	31.485	10.363	0.990
	50	5.511	15.600	752.968	5336.196	48.267	14.992	0.993
	60	5.858	9.698	377.393	5333.766	38.915	14.999	0.990
HVD 55 °C	30	7.352	50.190	1277.306	1.815 × 10^4^	25.449	4.409	0.985
	40	5.876	18.000	778.471	7273.839	43.248	10.998	0.991
	50	4.255	18.021	530.068	6427.004	29.414	12.447	0.988
	60	3.671	9.550	512.969	4199.839	53.714	19.048	0.992
HVD 65 °C	30	9.741	65.245	2054.107	2.380 × 10^4^	31.483	3.361	0.987
	40	7.926	39.079	1085.328	1.463 × 10^4^	27.773	5.467	0.987
	50	7.549	28.880	1011.784	9554.119	35.034	8.373	0.991
	60	6.956	22.999	957.637	5738.029	41.638	13.942	0.994
HVD 75 °C	30	7.816	57.858	1335.159	1.856 × 10^4^	23.076	4.311	0.986
	40	6.288	30.236	975.934	1.119 × 10^4^	32.277	7.149	0.988
	50	5.263	18.040	602.605	8122.873	33.404	9.849	0.988
	60	4.896	20.384	621.951	7975.713	30.512	10.030	0.988

**Table 3:** WLF constants and Arrhenius activation energy for maize kernels (stress relaxation).

Equation	Parameters	Drying Condition					
		ND	HVD 35 °C	HVD 45 °C	HVD 55 °C	HVD 65 °C	HVD 75 °C
WLF	*C*_1_	14.990	5.995	8.406	7.174	7.758	5.882
	*C*_2_ (K)	191.700	56.655	134.400	72.001	71.984	43.807
	*R* ^2^	0.997	0.999	0.953	0.992	0.986	0.857
Arrhenius	*E_a_*	126.900	124.200	90.860	117.300	126.600	134.800
	*R* ^2^	0.997	0.959	0.934	0.967	0.960	0.963

**Table 4:** WLF constants and Arrhenius activation energy for maize kernels (creep).

Equation	Parameters	Drying Condition					
		ND	HVD 35 °C	HVD 45 °C	HVD 55 °C	HVD 65 °C	HVD 75 °C
WLF	*C*_1_	6.127	32.020	2.226	15.873	4.435	9.671
	*C*_2_ (K)	17.771	257.500	8.346	73.297	13.647	40.668
	*R* ^2^	0.998	0.963	0.985	0.998	0.966	0.989
Arrhenius	*E_a_*	250.599	220.498	111.035	292.645	199.302	255.960
	*R* ^2^	0.932	0.945	0.870	0.987	0.936	0.955

**Table 5:** Equation parameters for long-period stress relaxation of maize kernels.

Equation	Parameters	Drying Condition					
		ND	HVD 35 °C	HVD 45 °C	HVD 55 °C	HVD 65 °C	HVD 75 °C
Generalized Maxwell equation	*E*_1_ (MPa)	4.449	2.543	1.118	2.326	2.297	1.753
	*E*_2_ (MPa)	2.802	1.796	1.674	3.555	2.763	3.166
	*E*_3_ (MPa)	2.858	2.573	2.923	4.050	3.253	2.485
	*τ*_1_ (s)	2177.795	4704.469	55.148	8945.749	5866.535	8.865 × 10^5^
	*τ*_2_ (s)	123.826	1.676 × 10^5^	2.760 × 10^4^	1.604 × 10^5^	2.716 × 10^5^	242.832
	*τ*_3_(s)	8.286 × 10^4^	155.342	821.170	143.129	146.080	2.284 × 10^4^
	*E*_0_ (MPa)	0.668	0.095	0.095	0.322	0.172	0.365
	*R* ^2^	0.994	0.980	0.990	0.992	0.987	0.996
Kohlrausch–Williams–Watts (KWW) equation	*E*_0_ (MPa)	16.501	10.670	7.847	13.554	14.006	12.593
	*τ*	1059.509	958.363	798.541	3888.804	791.291	1602.291
	*β*	0.194	0.191	0.271	0.187	0.149	0.145
	*R* ^2^	0.992	0.978	0.984	0.974	0.978	0.995

**Table 6:** Equation parameters for long-period creep of maize kernels.

Equation	Parameters	Drying Condition					
		ND	HVD 35 °C	HVD 45 °C	HVD 55 °C	HVD 65 °C	HVD 75 °C
Burgers equation	*E*_1_ (MPa)	10.244	6.968	5.251	6.081	8.161	6.503
	*E*_2_ (MPa)	17.795	13.754	11.929	7.081	18.004	11.459
	*η*_2_ (MPa s)	4.317 × 10^5^	2.145 × 10^4^	3.291 × 10^4^	1.142 × 10^5^	1.789 × 10^5^	1.821 × 10^5^
	*η*_1_ (MPa s)	3.389 × 10^7^	4.294 × 10^6^	3.126 × 10^5^	6.964 × 10^7^	8.018 × 10^6^	4.386 × 10^7^
	*R* ^2^	0.951	0.956	0.966	0.950	0.946	0.922
Two-parameter power law equation	*a*	0.005	0.008	0.010	0.008	0.006	0.009
	*b*	0.088	0.083	0.097	0.088	0.080	0.067
	*R* ^2^	0.905	0.932	0.879	0.970	0.912	0.970
Findley power law equation	*a*	2.418 × 10^−4^	0.001	2.829 × 10^−4^	0.003	4.020 × 10^−4^	0.003
	*b*	0.263	0.254	0.392	0.148	0.254	0.113
	*ε*_0_	0.007	0.010	0.014	0.008	0.008	0.007
	*R* ^2^	0.978	0.977	0.974	0.982	0.974	0.976

## Data Availability

Data are contained within the article.
